# Sick Leave Patterns in Common Rheumatological Diseases

**DOI:** 10.7759/cureus.34034

**Published:** 2023-01-21

**Authors:** Ibtisam M Jali

**Affiliations:** 1 Internal Medicine and Rheumatology Department, King Abdulaziz University Hospital, Jeddah, SAU

**Keywords:** saudi, burden, low back pain, osteoarthritis, rheumatoid arthritis, sick leave

## Abstract

Objectives

The study aims to analyze the patterns and determinants of sick leaves (SL) associated with the most common rheumatological diseases and estimate the associated productivity loss cost (PLC).

Method

A retrospective study reviewed all SLs that were issued from a rheumatology outpatient clinic between 2016 and 2019 for the following diagnoses: low back pain (LBP), knee osteoarthritis (OA), rheumatoid arthritis (RA), and disc disorders. The duration of each sick leave was captured and analyzed by patients’ age category, gender, and diagnosis. The human capital method was used to estimate the PLC.

Result

One thousand and two SLs have been issued during the study period, for a cumulative 4,649 days. The majority of the patients were female (86.3%), and the mean (SD) age was 42.01 (10.71) years. SL durations ranged from 2 to 14 days. The most frequent diagnosis was RA (34.3%), followed by LBP (30.1%). Disc disorder, knee OA, and RA were independently associated with 2.01 (p=0.014), 9.07 (p<0.001), and 7.75 (p<0.001) odd ratios for long SL (≥7 days), by reference to LBP. The average PLC was estimated at $235.29 per day of sick leave, for a total cumulative cost of $235,755.30.

Conclusion

Rheumatological diseases are responsible for approximately 4.5 days of SL prescribing per day in our clinic, with an average yearly cost of $58,938.83. Monitoring the pattern of sick leave and identifying the interplay between their cofactors are essential to developing a comprehensive approach to enable evidence-based clinical practices along with advancements in work-based protective measures and policies.

## Introduction

Sick leave (SL) refers to paid days off due to medical reasons, allowing employees job security by avoiding job separation [1]. There has been a growing prevalence of sick leave since the establishment and promotion of worker’s rights globally [2]. This resulted in an increasing economic burden, which has been the focus of several studies in the last decades. In the first trimester of 2020, there was an ‘unprecedented’ increase in sickness absences, reaching about 116% in a large Spanish study [3].

Rheumatic and musculoskeletal disorders (RMDs) are considered the leading cause of medically justified absenteeism, contributing to substantial costs. This group of diseases, represented mainly by rheumatoid arthritis (RA), low back pain (LBP), chronic osteoarthritis (OA), and disc disorder radiculopathy, has a strong relationship with work skipping. A single-center study from the Netherlands reported that musculoskeletal diseases accounted for 39.1% of sick leaves, followed by cardiovascular (12.7%), respiratory (11.6%), and mental (7.9%) diseases [4]. With the same conclusion, a multicohort study reported that musculoskeletal diseases were the most common diagnoses for sick leave after 3.8-6.7 years of follow-up [5]. This reflects a significant economic burden. In a study from the USA, the estimated national indirect costs of RA-induced absenteeism were $252 million per annum [6]. Similarly, other data reported that LBP led per se to an estimated sick leave expenditure of €244.7 million in the Dutch workforce [7]. Furthermore, a real-world study stated that 16.9% of patients with OA pain took at least six months of sick leave. The same study estimated the cost of illness due to absenteeism among OA active workers as €2,594 per patient per year [8].

Although workers with RMDs have an increased risk of taking sick leave, this risk may vary between the different diagnoses as well as between individuals due to other factors such as sex, age, and comorbidity. Women, older workers, and patients with RA and LBP have a higher frequency of sick leave requests. These factors may also determine the duration of absenteeism episodes [4,6,9,10]. More specific characteristics of individuals with RMDs have been explored, such as lifestyle, substance use, smoking and drinking status, BMI, and physical activity, to establish their impact on interrupted workday prevalence [5].

Identifying the patterns of sick leave in RMDs is of paramount importance for the planning of treatment and rehabilitation, as well as for the evaluation of the prognosis to improve the management approaches. Additionally, revealing the impact of non-modifiable factors, such as gender and age, enables the promotion of occupational health equity programs in workplaces to support disadvantaged workers and those who are at high risk of experiencing work-related health problems [11]. Finally, further preventive interventions may be directed towards the modifiable or avoidable, occupational and non-occupational factors of recurrence or extension of sick leaves.

We carried out the present study to analyze the patterns of sick leaves associated with the most common RMDs in a tertiary care center and to determine factors associated with sick leave duration.

## Materials and methods

Design and setting

This was a retrospective chart review that was conducted at the Outpatient Rheumatology Clinics of King Abdulaziz University (KAU) Hospital, Jeddah, Saudi Arabia. The study protocol was reviewed and ethically approved by the institutional review board of KAU (Ref# 618-20).

Population

The study included all sick leaves issued from the rheumatology clinics between 1 January 2016 and 31 December 2019 for the following diagnoses: LBP, knee OA, RA, and disc disorders.

Data collection

We searched the hospital information system for all electronic records comprising sick leaves, which were sorted by date and issuing department. The eligible sick leaves were selected, and the corresponding patients’ numbers were used to capture the main demographic and clinical data, including gender, age, and diagnosis. Additionally, the sick leave duration was collected at the same time, and all these data were captured in an Excel sheet.

Estimation of the productivity loss

To estimate the economic cost of sick leaves, the human capital method was used to measure the potential value of productivity loss due to sick leave [12]. However, since the data lacked the participants’ wages or professional categories, we used the GDP per capita as a proxy for the wages, assuming that each absenteeism period costs its equivalent of GDP per capita adjusted for the duration. The Saudi GDP per capita for the years 2016-2019 was $19,878.8 (2016), $20,802.5 (2017), $23,337 (2018), and $23,139.8 (2019) [13]. Additionally, the days of activity lost for the weekend off-days were adjusted by subtracting one day out of each consecutive six days, two days out of each consecutive 7-12 days, three days out of each consecutive 13 days, and four days out of each consecutive 14 days. The number of adjusted days was then multiplied by the GDP per capita per day for the corresponding year. However, since the year of sick leave was not collected, the average GDP per capita for the four years 2016-2019 was used for all cases. Hence, the productivity loss cost (PLC) was estimated using the following formula:

PLC = adjusted no. days * 59.70$

Statistical methods

The database was cleaned and coded in an Excel sheet, then transferred to SPSS, version 21 for Windows (SPSS, Inc., Chicago, IL, USA), for statistical analysis. Descriptive statistics were carried out to analyze the patterns of sick leaves; categorical variables were presented as frequency and percentage, while continuous variables were presented as mean ± standard deviation (SD) with range. An independent t-test was used to compare the mean sick leave duration between genders, and a one-way analysis of variance (ANOVA) was used to compare the variance of sick leave duration between age groups and diagnoses. One-way ANOVA was completed with post hoc analysis for pairwise analysis; results are presented as the mean pairwise difference with the corresponding standard error (SE). Sick leave duration was dichotomized into short (<7 days) versus long (≥7 days), and the Chi-square test was used to analyze the associated factors. Multivariate logistic regression was carried out to analyze predictors of previously defined long sick leave defined (≥7 days). A p-value of <0.05 was considered to reject the null hypothesis.

## Results

Participants’ characteristics

One thousand and two sick leaves have been issued between January 2014 and December 2020. The majority of the patients were female (86.3%), and the mean (SD) age was 42.01 (10.71) years. The most frequent diagnosis was RA (34.3%), followed by non-specific LBP (30.1%), knee OA (21.7%), and disc disorders (13.9%), contributing to a cumulative total of 4,649 days of sick leave. Sick leave duration ranged from two to 14 days, with a mean (SD) of 4.64 (2.86) (Table 1).

**Table 1:** Participant’s characteristics (N=1002)

Parameter	Level	Frequency	Percentage	Mean	SD
Gender	Male	137	13.7		
	Female	865	86.3		
Age (years)				42.01	10.71
	Range			13	64
Age category (year)	<20	55	5.5		
	20–29	71	7.1		
	30–39	224	22.4		
	40–49	390	38.9		
	50+	262	26.1		
Diagnosis	Rheumatoid arthritis	344	34.3		
	Low back pain	302	30.1		
	Knee osteoarthritis	217	21.7		
	Disc disorders	139	13.9		
SL duration (days)				4.64	2.86
	Range			2	14

Estimated productivity loss cost

**Figure 1:**
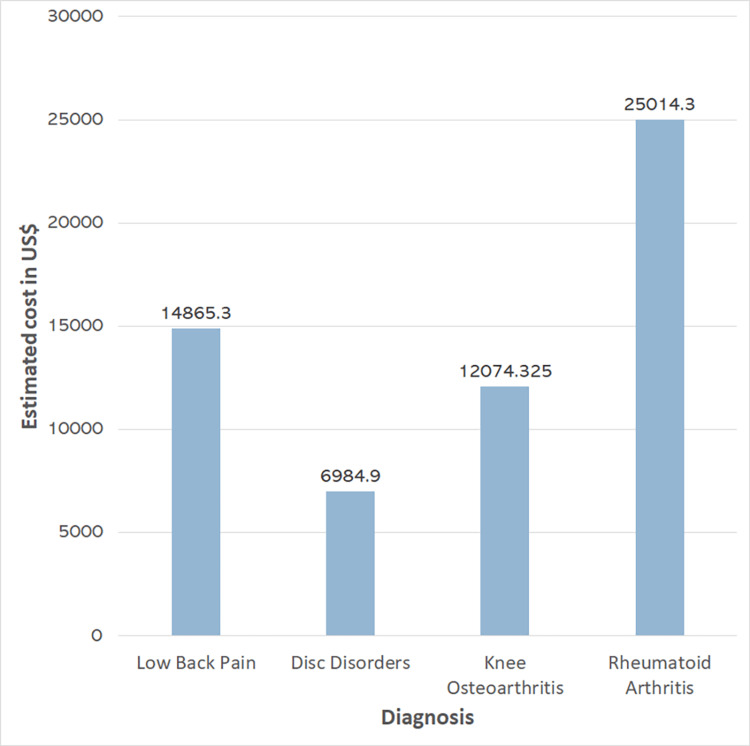
Yearly productivity loss costs due to sick leave for the most common rheumatological diseases, as estimated using a GDP per capita-based human capital model.

The average PLC was estimated at $235.29 (SD = $113.83) per sick leave, for a total cumulative cost of $235,755.30. This corresponds to a yearly cost of $58,938.83, distributed as follows: RA ($25,014.3), LBP ($14,865.3), knee OA ($12,074.33), and disc disorders ($6,984.9) (Figure 1).

Sick leave duration by gender, age, and diagnosis

Table 2 depicts the mean (SD) sick leave duration, contributed number of sick leave days, and percentage of sick leaves <7 versus ≥7 days by gender, age group, and diagnosis. The mean (SD) sick leave duration was longer in males (5.09 [2.85] days) versus females (4.57 [2.86] days), and the difference was statistically significant (p=0.049).

**Table 2:** Sick leave duration by gender, age, and diagnosis *Statistically significant result (p<0.05). Test used: t independent t-test; ^A^One-way ANOVA; ^X^Chi squared test.

Parameter	Level	Sick leave duration			Contributed no. days	Sick leave duration (%)		
		Mean	SD	p-value		<7 days	≥7 days	p-value
Gender	Male	5.09	2.85		697	57.7	42.3	
	Female	4.57	2.86	0.049*^t^	3953	68.7	31.3	0.011*^X^
Age (years)	<20	4.29	2.10		236	63.6	36.4	
	20–29	5.97	3.07		424	47.9	52.1	
	30–39	4.55	3.23		1019	74.1	25.9	
	40–49	4.58	2.88		1786	68.7	31.3	
	50+	4.52	2.50	0.002*^A^	1184	64.9	35.1	0.001*^X^
Diagnosis	Rheumatoid arthritis	5.97	3.40		2054	51.5	48.5	
	Low back pain	3.52	2.24		1063	88.7	11.3	
	Knee osteoarthritis	4.65	2.35		1009	53.9	46.1	
	Disc disorders	3.77	1.82	<0.001*^A^	524	79.9	20.1	<0.001*^X^

Regarding age, the age category of 20-29 years had the longest sick leave compared to the other age categories (p=0.002), with 52.1% of sick leaves being ≥7 days compared with 36.4% or less in the other age categories (p=0.001) (Table 2). Post hoc pairwise analyses confirmed that the age category 20-29 years was associated with a significantly longer sick leave duration, while no statistically significant difference was observed between the other age categories with one another (Table 3).

**Table 3:** Post hoc analysis *Statistically significant result (p<0.05). Test used: Tukey's honestly significant difference test.

Category (i)	Category (j)	Mean difference (i-j)	SE	p-value
Diagnosis				
Rheumatoid arthritis	Low back pain	2.44	0.21	
	Knee osteoarthritis	1.32	0.23	
	Disc disorders	2.20	0.27	
Low back pain	Rheumatoid arthritis	−2.44	0.21	
	Knee osteoarthritis	−1.13	0.24	
	Disc disorders	−0.25	0.27	0.805
Knee osteoarthritis	Rheumatoid arthritis	−1.32	0.23	
	Low back pain	1.13	0.24	
	Disc disorders	0.88	0.29	0.013*
Disc disorders	Rheumatoid arthritis	−2.20	0.27	
	Low back pain	0.25	0.27	0.805
	Knee osteoarthritis	−0.88	0.29	0.013*
Age category				
<20	20–29	−1.68	0.51	0.009*
	30–39	−0.26	0.43	0.975
	40–49	−0.29	0.41	0.957
	50+	−0.23	0.42	0.982
20–29	<20	1.68	0.51	0.009*
	30–39	1.42	0.39	0.002*
	40–49	1.39	0.37	0.001*
	50+	1.45	0.38	0.001*
30–39	<20	0.26	0.43	0.975
	20–29	−1.42	0.39	0.002*
	40–49	−0.03	0.24	1.000
	50+	0.03	0.26	1.000
40–49	<20	0.29	0.41	0.957
	20–29	−1.39	0.37	0.001*
	30–39	0.03	0.24	1.000
	50+	0.05	0.23	0.999
50+	<20	0.23	0.42	0.982
	20–29	−1.45	0.38	0.001*
	30–39	−0.03	0.26	1.000
	40–49	−0.05	0.23	0.999

Rheumatoid arthritis contributed the longest sick leave duration with a mean (SD) = 5.97 (3.40) days for a cumulative total of 2,054 days, and 48.5% of sick leaves were ≥7 days, which was significantly greater than other diagnoses (p<0.05) (Table 2). Post hoc pairwise analyses showed no statistically significant difference in mean (SD) sick leave duration between low back pain and disc disorders (p=0.805) (Table 3).

Predictors for long sick leave

The multivariate logistic model showed that diagnosis was an independent factor for sick leave duration, with disc disorder, knee osteoarthritis, and rheumatoid arthritis being associated with 2.01 (p=0.014), 9.07 (p<0.001), and 7.75 (p<0.001) odd ratios of long sick leave (≥7 days), by reference to low back pain (Table 4).

**Table 4:** Predictors for long sick leave in rheumatological diseases OR: odds ratio, CI: confidence interval. *Statistically significant result (p<0.05).

Predictor	Level	OR	95%CI		p-value
Gender	Male	1.47	0.98	2.23	0.065
	Female	Ref	-	-	-
Age (years)	<20	1.18	0.59	2.37	0.641
	20–29	1.71	0.93	3.16	0.085
	30–39	Ref	-	-	0.017*
	40–49	0.71	0.46	1.10	0.124
	50+	0.65	0.40	1.05	0.078
Diagnosis	Low back pain	Ref	-	-	
	Disc disorders	2.01	1.15	3.49	0.014*
	Knee osteoarthritis	9.07	5.49	14.98	
	Rheumatoid arthritis	7.75	5.05	11.90	

## Discussion

Summary of findings

This four-year retrospective study explored the patterns of sick leave in the most common RMDs and analyzed the baseline clinical and demographic factors and predictors for sick leave duration. Nonspecific LBP and RA represented were the main diagnoses responsible for sick leaves in this cohort, contributing a cumulative 2,054 and 1,063 days of absenteeism, respectively. The sick leave duration was significantly longer among females, the middle-aged group (20-29 years), and patients with RA and knee OA. Furthermore, disc disorder, knee OA, and RA were independently associated with 2.01 (p=0.014), 9.07 (p<0.001), and 7.75 (p<0.001) odd ratios of long sick leave (≥7 days), by reference to LBP. Finally, using the available data, we estimated a $58,938.83 yearly PLC of sick leaves for all diagnoses, for an average of $235.29 per sick leave.

Relationship between work and sick leave in rheumatic diseases

Work-related stress and rheumatologic diseases have a bidirectional relationship, resulting in a conundrum of sick leave maintenance. Physical work intensity may interfere with the level of control and prognosis of RMDs by means of stress-mediated effects on proinflammatory and anti-inflammatory pathways, decreased adherence to long-term treatments, and psychological distress due to exhaustion, anxiety, work conflicts, etc. [14]. Reciprocally, poorly managed rheumatologic disorders could be a source of significant work disability and decrease workers’ productivity. This may be due to direct factors such as morning stiffness and the inability to use joints to make repetitive, high-magnitude movements, or indirect factors such as sleep disturbances [15,16]. This results in a continuous loop of absenteeism enhancers that can only be interrupted if interventions are simultaneously targeted at both contributing factors.

Patterns of sick leave by diagnosis

Rheumatoid arthritis was the most frequent diagnosis responsible for sick leave in our cohort. This is consistent with other research results. A retrospective study found that 67% of RA employees had sick leave versus 58% of those without RA, and out of all sick leave seekers, RA individuals missed more workdays per year than those without RA [6]. This may be expected given that RA is highly prevalent in comparison with other RMDs [17] and that RMDs are the most common cause of sick leave [4]. On the other hand, work-related physical and psychological stress may increase inflammatory biomarkers, which increase the risk of flares and functional disability in RA patients, leading to frequent absenteeism [14,18]. Yet, the exact mechanisms of this association are still to be uncovered. Furthermore, we observed that RA patients took the longest duration of sick leave per episode. Closely, a study found that disc disorders and RA had the longest periods of sick leave compared to the other common RMDs, with a mean of 147 days [19]. Occupational fatigue is a recurring problem in RA individuals and can lead to prolonged periods of disability with decreased work performance, which could motivate longer durations of rest [20,21].

Besides RA, nonspecific low back pain (i.e., in the absence of disc disorders) was the second most frequent diagnosis associated with sick leave. LBP is a common disorder among workers that motivates sick leave demands [10]. A study showed that 23.9% of rehabilitation professionals in Saudi Arabia had LBP-related sick leave [22]. Several professional sectors are exposed to high risks of LBP, such as industry, agriculture, forestry, and fisheries [23,24]. This is due to a wide range of movements being associated with the development of LBP, notably extreme trunk flexions, loads lifting, pushing or pulling, and vibrations, as well as extended vicious positions of the lumbar spine [25].

Demographic determinants of sick leave

Our study demonstrated a substantially high female ratio among the total sick leaves. Similarly, previous studies have raised concern about a significant difference in RMDs-related sick leave across genders [9]. This may be explained by the significant gender disparity of most rheumatic diseases, notably RA and LPB, which are more common in females [24,26]. Factors related to ergonomics and the work environment may also contribute to this difference. Additionally, women have more work-related stress, a lower occupational grade, higher home-work interference, and poorer social support during work, which are associated to long-term sick leaves [27,28]. Along with this, the psychological impact of occupational stress and job conflicts tends to be more deleterious in females [29]. In contrast, we observed that males had relatively longer absence periods per sick leave, which may be associated with the nature of male-dominated professions characterized by hard physical work. This may result in more severe back and joint pain and stiffness, which require longer recovery times [23]. Other evidence suggests that individuals who have decision-making and leadership responsibilities would be more likely to require full recovery before resuming work and that such positions often apply to males [28].

Age was another determinant for sick leave patterns. We observed an increased frequency of middle-aged individuals (40-49 years old) among the total sick leaves, while the 20-29-year age group had longer sick leave durations. Possible explanations for the first observation can be found in the social and professional features of the 40-49 age group. Generally, the forties coincide with a carrier phase characterized by more affirmed work positions, more family responsibilities, and greater social expectations, which may result in increased social and psychological pressure [30,31]. More specifically, women in their forties and fifties are in the peri-menopause period, in which many biological changes occur affecting mood, self-confidence, and motivation, ultimately leading to more psychological vulnerability and decreased performance [32-34]. Estrogen drops have been shown to accelerate lumbar disc degeneration, which may explain the increased prevalence of LBP after menopause [35,36]. In other terms, this is the age of "squeezed workers," on whom maximum attention and concern should be risen.

On the other hand, younger adults (20-29 years old) had longer sick leave per episode. A suggested explanation is that this age group is probably more involved in physically strenuous work such as lifting, bending, long-standing, and nightshifting, which would induce more severe pain and prolonged fatigue episodes. Additionally, rheumatic diseases, such as RA, are characterized by more debilitating attacks during the first years, coinciding with a younger age, and tend to become more tolerable over time after years of treatment [37,38].

Economic cost of sick leave in rheumatological diseases

Using the human capital model based on the GDP per capita, we estimated the yearly productivity loss for sick leaves as $58,938.83, while the average cost per sick leave was estimated at $235.29, regardless of the diagnosis. These estimates from a single center represent a portion of the economic burden of RMDs, as they only include the costs related to productivity days lost for sick leave, regardless of the other direct costs such as disability-adjusted life years (DALYs) with long-term cumulative costs. The indirect costs of RMDs due to loss of productivity are alarming worldwide. A global study found that musculoskeletal disorders caused 138.7 million DALYs in 2017 [39]. In another study, LBP, OA, and RA accounted for 606.05, 292.11, and 192.46 DALYs per 100,000 inhabitants, respectively [40]. In terms of productivity-adjusted life years (PALYs) and years lived with disability (YLD), OA, as an example, caused 1,943,287 PALYs and 118.8 YLD [41,42]. Although the method used to estimate the costs represents several flaws and limitations, notably the absence of relevant data, it provided an approximation of the economic burden resulting from sick leave.

Tackling the burden of sick leave in rheumatic diseases

In a holistic care approach, such data enables testing the cost-effectiveness of treatment and rehabilitation interventions to reduce the duration and prevent the recurrence of sick leaves. Furthermore, from the employers’ perspective, considerable cost savings may be achieved by the implementation of cost-effective interventions to improve work ergonomics and reduce work stress on diseased employees and those at high risk of developing rheumatological diseases. It has been demonstrated that ergonomic exposures at work, notably in certain professions, represent the major cause of LBP-related disability worldwide, resulting in approximately 22 million DALYS per year [43]. Several interventions may be implemented in workplaces to promote ergonomics and reduce the stress of physically demanding tasks. For example, employees with arthritis or other rheumatic diseases who benefit from work-based assistance in to use of ergonomics have seen their work efficiency improved and their work-related disability reduced [44].

From a clinical perspective, tackling the economic burden of sick leave depends on the appropriateness of the prescription in terms of indication and duration. Our findings showed that the cumulative number of sick leave days was 4,649 in one center, representing an average of 1,162.25 days per year. This means that approximately 4.5 days of sick leave are prescribed daily in our clinic, considering that the rheumatology clinic is open five days per week. Interventions to reduce unnecessary sick leave prescribing should target both requesters and prescribers. Patient education can be an effective strategy by providing information about the underlying disease and occupational health and safety precautions to reduce the risk of injury related to work. An educational back pain intervention led to a significant reduction in sick leave in a randomized controlled trial [45]. On a larger scale, raising public awareness about the economic costs of unnecessary sick leave may be effective in reducing requests. Likewise, physicians should be aware of the economic burden of sick leave on public health and the economy, thereby avoiding complacent prescribing. It was observed that insurance physicians were stricter in assessing sick leave than occupational physicians [46]. Similarly, clinical specialists were demonstrated to prescribe shorter sick leaves than general practitioners [47], while older physicians tended to authorize more days of sick leave than younger physicians [48]. This highlights the importance of the human factor in the heterogeneity of sick leave prescribing, indicating the absence of standardized, evidence-based clinical practice guidelines. Other data showed that, for patients with the same diagnosis, surgeons prescribed more sick leave for those having physical jobs than for those working in an office [49]. Although the latter approach appears to be applicable in the context of rheumatology, it requires an evidence-based implementation to consider the other factors.

Study limitations

The main limitation of the present study is the lack of data about the major factors and confounders of sick leave, such as the type of work, lifestyle parameters, comorbidities, and duration of the disease. Additionally, the economic data, such as wages or the working sector, would have enabled a more accurate estimation of the economic costs.

## Conclusions

Rheumatological diseases are responsible for approximately 4.5 days of sick leave prescribed per day in our clinic, with an average yearly cost estimated at $58,938.83. The majority of sick leaves concern women in their forties, and RA and LBP are the most frequent diagnoses. This emphasizes the need for medical and occupational interventions to improve the work environment for patients with RMDs, especially in high-risk subcategories. The quantification and analysis of direct and indirect costs of RMDs, including sick leave costs, are key to addressing the cost-effectiveness of such interventions. Monitoring the pattern of sick leave and identifying the interplay between their cofactors would help to develop a comprehensive approach to enable standardized and evidence-based practices along with advancements in work-based protective measures and policies. The same advancements would enable achieving a reduction in the frequency and duration of sick leaves, which will ultimately reduce both the health and economic burden of RMDs. From a clinical perspective, the role of physicians’ awareness and patient education remains central.
